# Adaptive Evolution of the *Chlamydia trachomatis* Dominant Antigen Reveals Distinct Evolutionary Scenarios for B- and T-cell Epitopes: Worldwide Survey

**DOI:** 10.1371/journal.pone.0013171

**Published:** 2010-10-05

**Authors:** Alexandra Nunes, Paulo J. Nogueira, Maria J. Borrego, João P. Gomes

**Affiliations:** 1 Department of Infectious Diseases, National Institute of Health, Lisbon, Portugal; 2 Department of Epidemiology, National Institute of Health, Lisbon, Portugal; National Institutes of Health, United States of America

## Abstract

**Background:**

*Chlamydia trachomatis* is one of the most disseminated human pathogens, for which no vaccine is available yet. Understanding the impact of the host pressure on pathogen antigens is crucial, but so far it was only assessed for highly-restricted geographic areas. We aimed to evaluate the evolutionary picture of the chlamydial key antigen (MOMP), which is one of the leading multi-subunit vaccine candidates, in a worldwide basis.

**Methodology/Principal Findings:**

Using genetics, molecular evolution methods and mathematical modelling, we analyzed all MOMP sequences reported worldwide, composed by 5026 strains from 33 geographic regions of five continents. Overall, 35.9% of variants were detected. The evolutionary pattern of MOMP amino acid gains/losses was found to differ from the remaining chromosome, reflecting the demanding constraints of this porin, adhesin and dominant antigen. Amino acid changes were 4.3-fold more frequent in host-interacting domains (*P*<10^−12^), specifically within B-cell epitopes (*P*<10^−5^), where 25% of them are at fixation (*P*<10^−5^). According to the typical pathogen-host arms race, this rampant B-cell antigenic variation likely represents neutralization escape mutants, as some mutations were previously shown to abrogate neutralization of chlamydial infectivity *in vitro*. In contrast, T-cell clusters of diverse HLA specificities are under purifying selection, suggesting a strategy that may lead to immune subversion. Moreover, several silent mutations are at fixation, generating preferential codons that may influence expression, and may also reflect recombination-derived ‘hitchhiking-effect’ from favourable nonsilent changes. Interestingly, the most prevalent *C. trachomatis* genotypes, E and F, showed a mutation rate 22.3-fold lower than that of the remainder (*P*<10^−20^), suggesting more fitted antigenic profiles.

**Conclusions/Significance:**

Globally, the adaptive evolution of the *C. trachomatis* dominant antigen is likely driven by its complex pathogenesis-related function and reflects distinct evolutionary antigenic scenarios that may benefit the pathogen, and thus should be taking into account in the development of a MOMP-based vaccine.

## Introduction

*Chlamydia trachomatis* is an obligate intracellular pathogen that causes ocular-genital infections in humans. Trachoma (chlamydial genotypes A–C and Ba) is the world's leading cause of preventable blindness with special impact in resource-poor nations, which has been recently placed on the WHO's priority list for intervention [1]. Also, the asymptomatic character of most genital chlamydial infections (genotypes D–K, Da, Ia, Ja and L1–L3) makes this pathogen the major cause of bacterial sexually transmitted infections worldwide [2]. Thus, *C. trachomatis* constitute a major public health problem, and the development of effective preventive strategies, such as a vaccine, are urgently needed. So far, vaccine attempts failed to provide broad coverage and conferred limited protection [3], [4].

One of the leading multi-subunit vaccine candidates is the *C. trachomatis* major outer membrane protein (MOMP), coded by *ompA*, whose variations underlie strain classification into serogroups (B, C and Intermediate) or genotypes [5]. It is the dominant antigen with tenths of well-defined species and serovar-specific epitopes, eliciting both the humoral and cellular immune responses [3], [6]–[9]. MOMP constitutes about 60% of the membrane dry-weight [10] and is a trimer stable under reducing conditions [11]. Each monomer is predicted to form a 16-stranded β-barrel structure resembling a porin [12], where the four highly variable domains (VDI to VDIV) of the protein are surface exposed. Also, the charge properties of VDs support an adhesion role for MOMP [13], both at the time of attachment to the host cell as well as to the host inclusion membrane to interact with the mitochondria and endoplasmatic reticulum [14], [15]. Moreover, this protein is likely an important virulence factor as it has been demonstrated that subtle mutations may yield profound distinct strain-specific neutralizing antibody responses in a nonhuman primate trachoma model [16], and it presents an immune decoy function by blocking the binding of broadly protective species-common pan-neutralizing antibodies [17].

It is believed that *C. trachomatis* evolution involved the loss of huge portions of its genome upon becoming an obligate intracellular parasite [18]. Subsequently, the evolutionary accumulation of mutations and insertion/deletion (indel) events resulted in the formation of pseudogenes [19], [20], the phylogenetic segregation of strains according to their cell-appetence [21], [22], and the generation of specific evolutionary pictures for heterogeneous loci categories [21]. Recently, a geographic-restricted study showed an adaptation of the *C. trachomatis* key antigen to the host immune pressure [23], shedding some lights about the MOMP adaptive evolution. Here, we aimed to determine MOMP evolutionary picture in a worldwide basis by using all available *ompA* sequences reported in the literature, which totalize 5026 strains isolated in 33 distinct geographic regions dispersed by five continents. We mapped MOMP positions that are more prone to change as well as specific mutations in epitopes that are at fixation around the globe. Globally, the adaptive evolution of the *C. trachomatis* dominant antigen likely reflects its complex pathogenesis-related function as an adhesion, porin, and major antigen, showing distinct evolutionary scenarios for B- and T-cell epitopes. The worldwide coverage of this assessment is unprecedented to date.

## Results

### Characterization of *C. trachomatis* specimens

A total of 5026 *C. trachomatis* strains isolated in 33 distinct geographic regions from five continents were analyzed in this worldwide survey. These encompassed all *ompA* genotypes with exception of L3, which was not found. Genotype E was the most prevalent type (30.0%), followed by genotypes F (12.9%), and L2 (9.8%). Globally, 1802 (35.9%) specimens were found to present *ompA* variant sequences when compared to that of the respective prototype strain (that define each *ompA* genotype), which have been used worldwide as comparative-baseline since their isolation up to 60 years ago (Table 1). Genotypes L2 and G presented the highest number of *ompA* variant specimens (n = 462 and n = 309, respectively), while genotypes E and F, which together represent almost half (42.3%) of all analyzed specimens, were the least variable, where only 83 out of 1464 E strains (5.6%) and 40 out of 640 F strains (6.3%) were variants. In fact, these two most worldwide prevalent types showed an *ompA* mutation rate 22.3-fold lower than that of the other genotypes (*P*<10^−20^), which suggest that the dominant antigenic profile of E and F MOMP may be better fitted to deal with the host immune system, which consequently may favor their ecological success.

**Table 1:** Description of the 17 baseline prototype strains representing the *C. trachomatis* genotypes.

Genotype/Strain	Year	Location	Biological Sample	GenBank Accession No.
A/Har13	1958	Egypt	Conjunctiva	NC007429
B/TW5	1959	Taiwan	Conjunctiva	AF304856
C/TW3	1959	Taiwan	Conjunctiva	AF352789
D/UW3	1965	Washington	Cervix	AE001338
Da/TW448	1985?	Taiwan	Conjunctiva	X62921
E/Bour	1959	California	Conjunctiva	X52557
F/IC-Cal3	1960?	California	Conjunctiva	X52080
G/UW57	1971	Washington	Cervix	AF063199
H/UW43	1965	Washington	Cervix	X16007
I/UW12	1966	Washington	Urethra	AF063200
Ia/IU4168	1987	Indiana	Urethra	AF063201
J/UW36	1971	Washington	Cervix	AF063202
Ja/IUA795	1986	n.a.	Cervix	AF063203
K/UW31	1973?	Washington	Cervix	AF063204
L1/440	1968	California	Lymph Nodes	M36533
L2/434	1968	California	Lymph Nodes	M14738
L3/404	1967	California	Lymph Nodes	X55700

**Figure 1:**
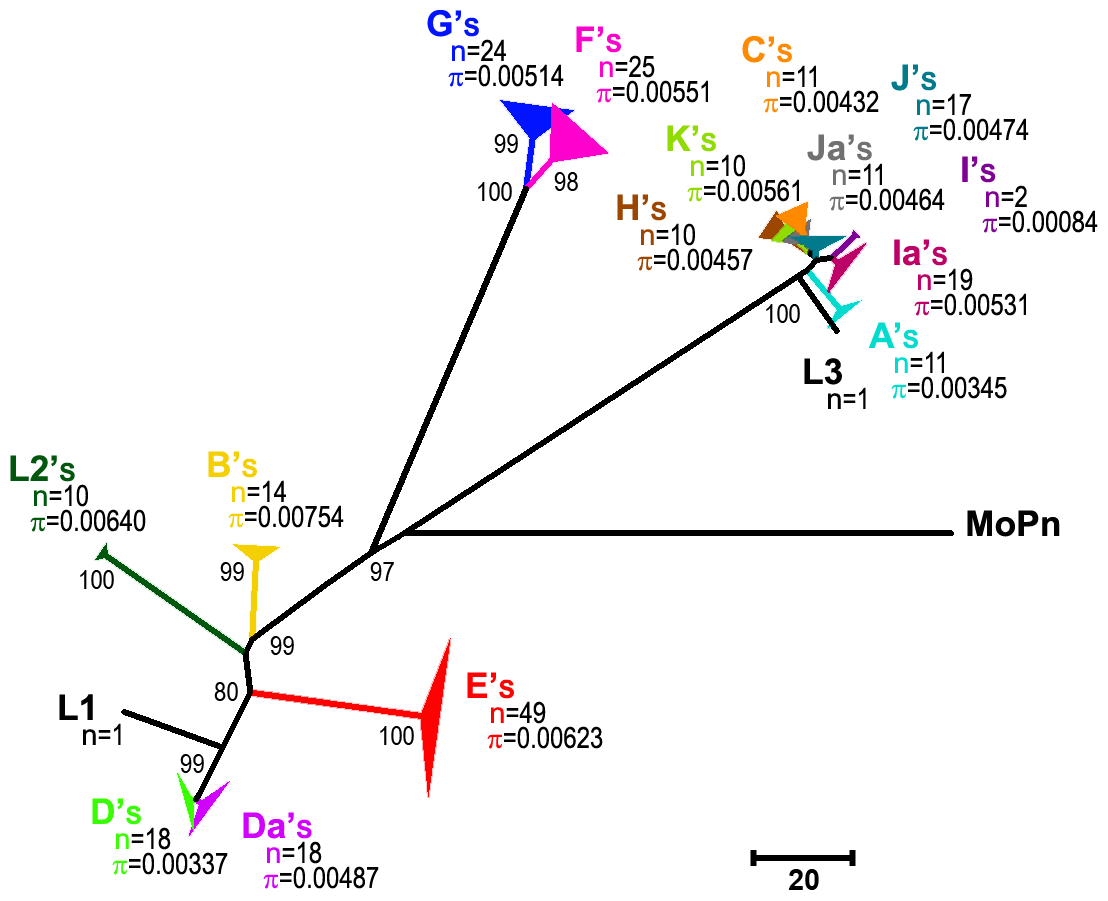
*ompA* phylogeny of the 251 *C. trachomatis* genetic variants. The represented tree was generated using the Neighbour-Joining method (with Kimura 2-Parameter model) but similar topologies were obtained with other methodologies (see Methods). Genotypes are represented in different colors. Thickness of solid triangles is proportional to the number of taxa from each genotype (n). The mean genetic diversity within each *ompA* genotype-population (π) is shown. The *ompA* sequence from *C. muridarum* strain MoPn (NC002620) was used to root the tree. Bootstrap values (1,000 replicates) are shown next to the branch nodes.

The 1802 variant specimens constitute 234 distinct types of genetic variants (i.e., with a distinct *ompA* mutational pattern that may be shared by several strains), for which no insertion/deletion or any trace of recombination event was found in *ompA*. Supporting this, robust phylogenies (Figure 1) were observed with maximum bootstrap support (97–100%) in the three main branches corresponding to the traditional chlamydial serogroups. In fact, nucleotide genetic distances among strains within the same genotype did not exceed 0.7% (standard error, SE 0.2%). In contrast to the other genotypes, most types of genetic variants for the least variable genotype E are represented by a single specimen, which suggests that the corresponding mutations are not being fixed.

### Worldwide *ompA* variability

**Figure 2:**
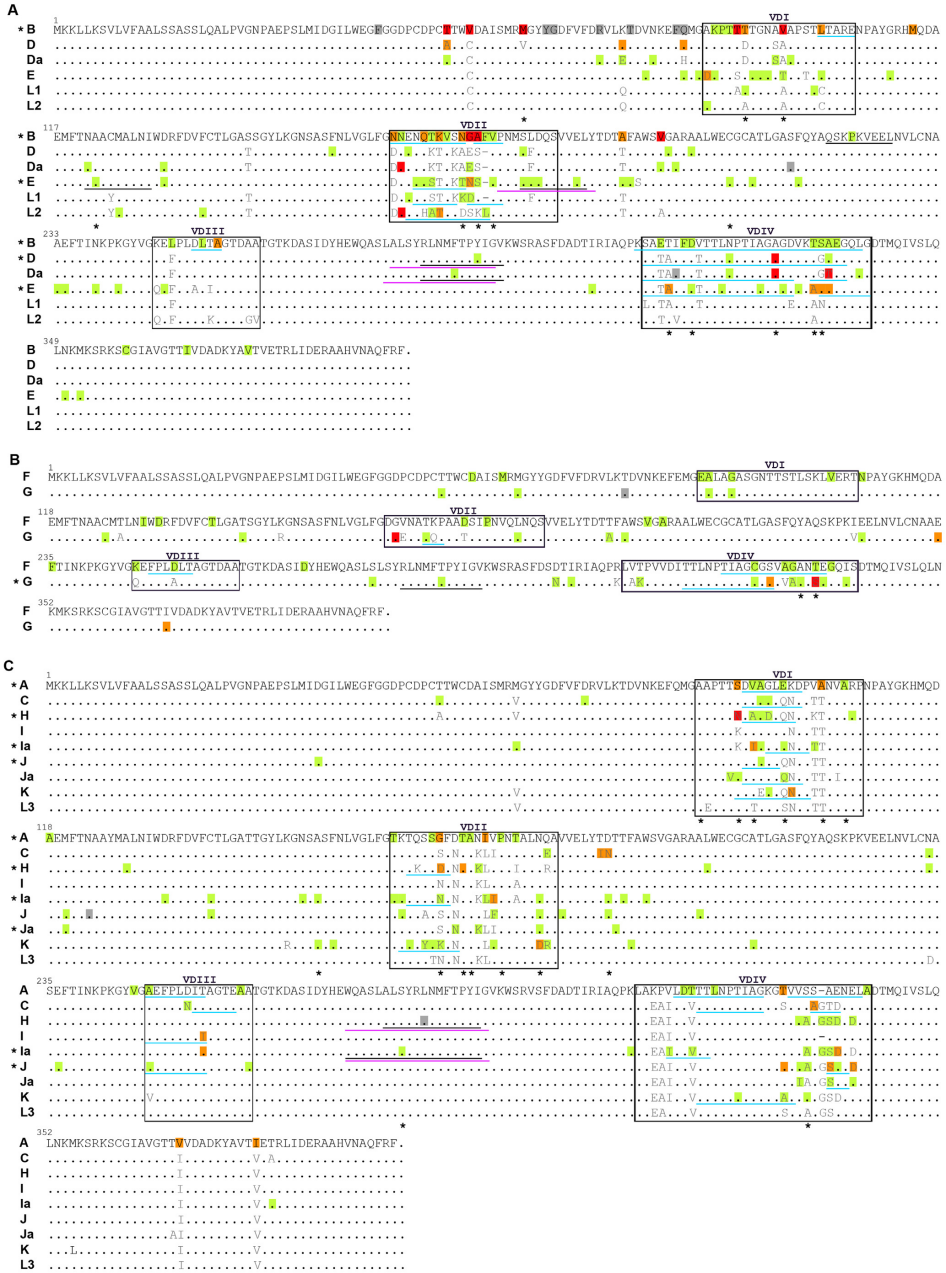
Worldwide mutational pattern of MOMP. Panels (A), (B), and (C) represent protein alignments of prototype strains according to serogroup B, Intermediate, and C, respectively. Amino acid changes that naturally occur among prototype strains are in grey characters (they do not represent changes among variant specimens and the respective prototype strains). Mutations resulting from the comparison between the 5026 strains isolated in 33 different geographic regions from five continents and the respective prototype strain are highlighted in different colors, corresponding to dissimilar degrees of evolutionary fixation. Green, orange and red represent amino acid changes occurring in strains isolated in a single, 2–4, and ≥5 geographic regions, respectively. Only silent mutations that occurred in strains isolated in ≥5 geographic regions are shown (highlighted in grey). Well-defined B-cell (blue), Th-cell (purple) and CTL (black) antigenic regions are underlined. Degenerated variable sites are marked with an asterisk.

Globally, a total of 511 variable sites (corresponding to the sum of all mutations between each type of genetic variants and the respective prototype strain) were found throughout *ompA*, where 56% of them occurred in VDs and 44% occurred in constant domains (CDs). About 68% of mutations yielded amino acid changes (with two thirds occurring in VDs), while ∼32% resulted in silent substitutions (with two thirds occurring in CDs). Figure 2 illustrates the most relevant variation found in MOMP for the 17 *C. trachomatis* genotypes, showing 70.3% (n = 359) of all variable sites found in the *ompA*. One fifth of them were exhibited by same-genotype strains isolated in two to 20 (for nonsynonymous mutations) or ≥5 (for silent mutations) different geographic regions (Figure 2), which may indicate a fixation of these mutations in those genotypes likely conferring them some structural or antigenic advantage. For example, the VDII Ala-to-Thr change occurring in the B-cell epitope ^172^AFVP^175^ [24] seems to become fixed within genotype B, as it was exhibited by 137 out of the 157 B variant specimens from 18 distinct geographic regions (data not shown). Moreover, of the 12 silent mutations that may be at fixation (Figure 2), 75% of them generated synonymous codons with high frequency usage in *C. trachomatis* [25]. For instance, for genotype J, the CDII C-to-T third-position change in the low frequent Asn codon AAC yielded the more frequent synonymous codon AAT (that is used almost two times more often than AAC) in 101 of the 113 (76%) J variant isolates from 10 distinct geographic regions (data not shown). Curiously, this AAT codon is conserved among all genotypes of B- and Intermediate-serogroups.

**Figure 3:**
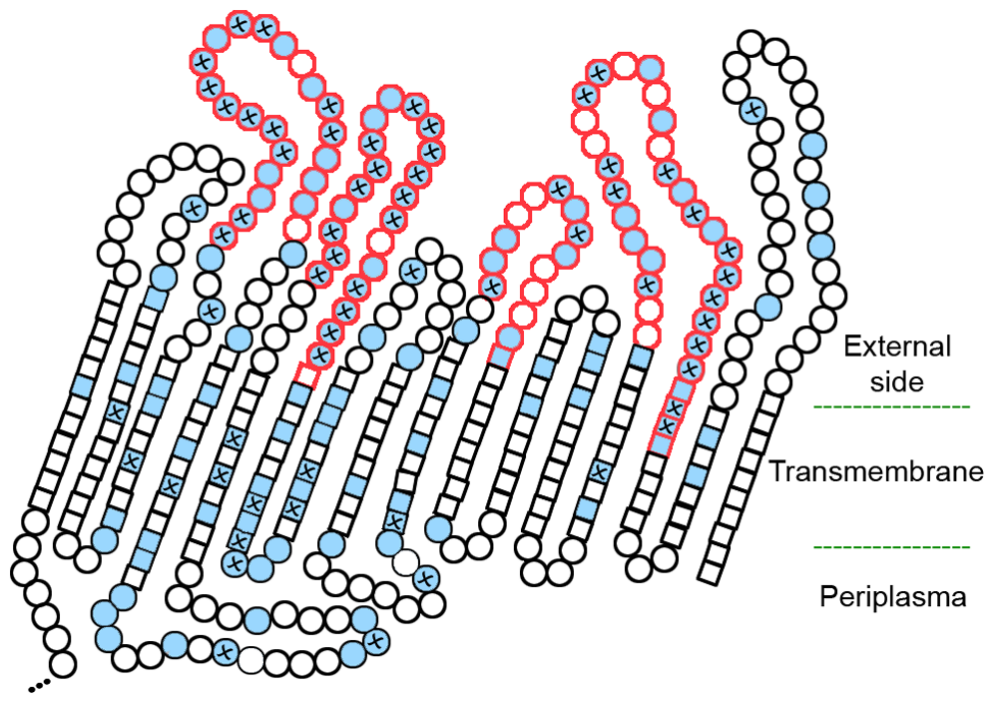
Topology sketch of MOMP mutational hotspots. VDs are presented in red, while CDs are in black. Circles represent amino acids located at external loops and periplasmic turns, while squares represent amino acids of the transmembrane β-strands (based on refs [12], [35]). Sites of the protein that were already subject to amino acid alterations are fulfilled in blue. Protein sites that were simultaneously mutated among strains from two to ten different genotypes (mutational ‘hotspots’) are marked with a cross.

In general, considering the MOMP genetic variability observed for all *C. trachomatis* genotypes, we found that 148 (37.3%) different sites of the protein were already subject to amino acid alterations (Figure 3). About 44% of the sites suffered mutations that occurred simultaneously among strains from two to ten different genotypes. Also, more than half of these ‘hotspot’ sites involved changes that seem to be at fixation, and were found to occur 2.4-fold more frequently within B-cell epitopes (*P*<10^−2^). Altogether, these results suggest that MOMP variability is not random as there are protein sites that are more prone to change, likely due to fitness advantages or simply to MOMP functional constraints.

### Evaluation of selective pressure on MOMP

To examine the selective pressure acting on MOMP, only the 234 types of genetic variants together with the 17 prototype strains were considered. This conservative approach avoids any bias arising from the existence of an unequal number of specimens represented by each type of genetic variant, resulting from the considerably different population sizes of the studies performed to date. The analysis of the molecular evolution of whole *ompA* sequence for all the 251 taxa showed a global mean ratio of synonymous (dS) to nonsynonymous (dN) substitution rates (dN/dS) of 0.26 (SE 0.03). The analysis *per* genotype showed dN/dS values >1 for genotypes A, G, Ia, and K (data not shown), suggesting a phenomenon of positive selection. However, due to the nondiscrimination of codons with distinct evolutionary signatures, this global dN/dS statistics may generate artifactual trends of synonymous and nonsynonymous rate variation when very few amino acids are under positive selection.

**Figure 4:**
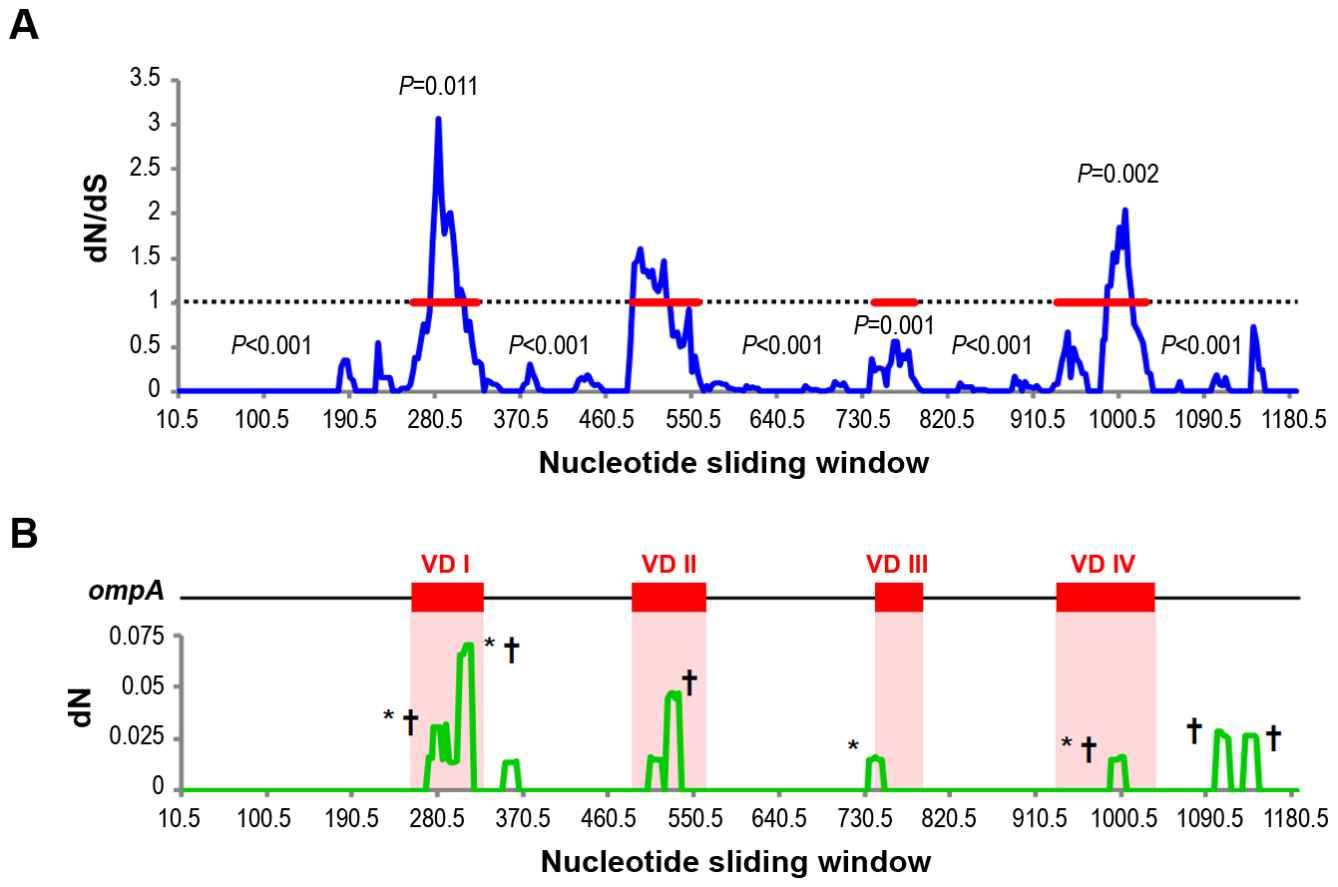
Selective pressure acting on *ompA*. (A) Ratio of dN/dS throughout *ompA*. dN/dS was estimated over a nucleotide sliding window (window size = 15; step size = 3), considering the 234 types of genetic variants and the 17 prototype strains. VDs are shown in horizontal red lines, while dashed line represents the traditional cut-off for neutral evolution. Only *P*-values showing statistical significance are shown. For positive and purifying selection *P*-values are displayed above and below the cut-off dashed line, respectively. Region containing MOMP CDIV species-specific cluster of five CTL and six Th epitopes (see text) is marked with (*). (B) dN values throughout *ompA* for genotype A (here, as an example). Calculations were performed over a sliding window (window size = 15; step size = 3), considering all types of genetic variants of genotype A. VDs (red boxes) display a global nonsynonymous rate 4.3-fold higher than that of the CDs (*P*<10^−12^). dN peaks involving mutations occurring within/adjacent to B-cell epitopes and/or mutations that are likely at fixation in this genotype are marked with (*) and (†), respectively.

In order to investigate if any portion of *ompA* is undergoing strong selection as well as which regions are not bound by strong functional constraints, a codon-based sliding-window analysis was also used to evaluate dN/dS throughout the entire gene. Although *ompA* showed a general trend of dN/dS<1, three distinct gene regions were identified with a statistically significant ratio>1 (Figure 4A), indicating that they are likely under positive selection. Interestingly, these regions are precisely located inside the VDI, VDII and VDIV, which (together with VDIII) showed a global nonsynonymous rate 4.3-fold higher than that of the CDs (*P*<10^−12^), suggesting a targeted amino acid variability in MOMP surface-exposed domains. In support of this, several putative positively selected codons were found within the VDII ^165^TQSSNF^170^ epitope or within a VDIV region containing multiple core B-cell epitopes for genotypes Ia and E, respectively. Almost all of these codons suffered nonsynonymous mutations that may lead to disruption of those epitopes in some E and Ia genetic variants. For all genotypes, almost all of the dN peaks observed occurred within or adjacent to well-defined MOMP epitopes, and also involved mutations that seem to become fixed among same-genotype strains (Figure 4B). Globally, it is possible that these results of positively selected regions may clearly be underestimated. In fact, it was recently demonstrated that for short time-scale models, the anticipated signature of adaptive evolution (dN/dS>1) is violated because dN/dS<1 was found to occur under both negative and positive selection [26].

### Is variability in MOMP a host evasion strategy?

The mutational trend in MOMP antigenic regions was investigated taking into account all B- and T-cell [cytotoxic T lymphocyte (CTL) and T helper (Th)] epitopes already described in the literature for the corresponding prototype strains (Figure S1). For this analysis, only minimal epitopes fully mapped were considered in order to avoid any bias arising from an overrepresentation of MOMP antigenic regions. Overall, of the total 511 mutations, 176 (34.4%) were found to occur within or adjacent to MOMP B- and T-cell epitopes with species, serogroup or genotype specificities. From these, 88.1% solely involved B-cell epitopes, which is statistically significant as they encompass only about 19% of the total protein length (*P*<10^−15^). We observed that 82.6% of them resulted in amino acid replacements, yielding a global nonsynonymous mutation rate almost threefold higher for these B-cell antigenic regions than for the rest of the *ompA* gene (*P*<10^−5^), which suggests targeted amino acid variability. More, 25% of these mutations were found to become worldwide fixed within some genotypes (*P*<10^−5^) (Figure 2). Also, some of them were previously shown to abrogate monoclonal antibody binding and neutralization of the infectivity of several prototype and/or variant strains *in vitro* [7], [8], [27]. For instance, the VDIV Ala-to-Thr change observed for genotypes D and Da was previously shown to prevent the antibody binding at the serogroup B ^323^IAGAG^327^ epitope for the known genovariant D^−^ [27]. This mutation seems to become fixed within genotypes D and Da, as it was found in strains from five and eight different geographic regions, respectively (Figure 2). Moreover, the VDI Ala-to-Val change found in 25% of the C variant strains from three distinct geographic regions was shown to prevent antibody binding to the ^92^DVAGL^96^ epitope (Figure 2), which is known to be highly intolerant to substitution [8]. Although the impact of these changes also depends on the epitope conformation that may vary among genotypes, the existence of such disrupting mutations in MOMP B-cell epitopes clearly evidence a *C. trachomatis* strategy to evade recognition and neutralization by host antibodies, allowing thereby the infection to evolve.

Of the 21 mutations occurred within or adjacent to T-cell antigenic regions, 66.7% involved clusters of CTL and Th epitopes. For example, several nonsynonymous mutations exhibited by D, Da, G, H, Ia and J specimens were found within the CDIV species-specific cluster of five human leukocyte antigen (HLA) class I-restricted minimal CTL epitopes [28] that superimposes (except for G) a cluster of at least six HLA class II-restricted core Th epitopes [29] (Figure 2). Another interesting example occurred for genotype E, where four nonsynonymous mutations were found within the E-specific CTL epitope ^177^SLDQSVVEL^185^ [28] that overlaps an E-specific Th-epitope-containing peptide [29], [30] and is adjacent to three well-defined B-cell core epitopes [7] (Figure 2A). Curiously, none of the above cited mutations seems to become fixed as they were exhibited solely by one strain of a single genotype or were restricted to a single geographic region, suggesting their nonfixation. In support of this, we found a statistically significant low dN/dS value for MOMP CDIV T-cell epitope region suggestive of purifying selection (Figure 4A).

### Evolutionary dynamics of amino acid gains and losses

**Figure 5:**
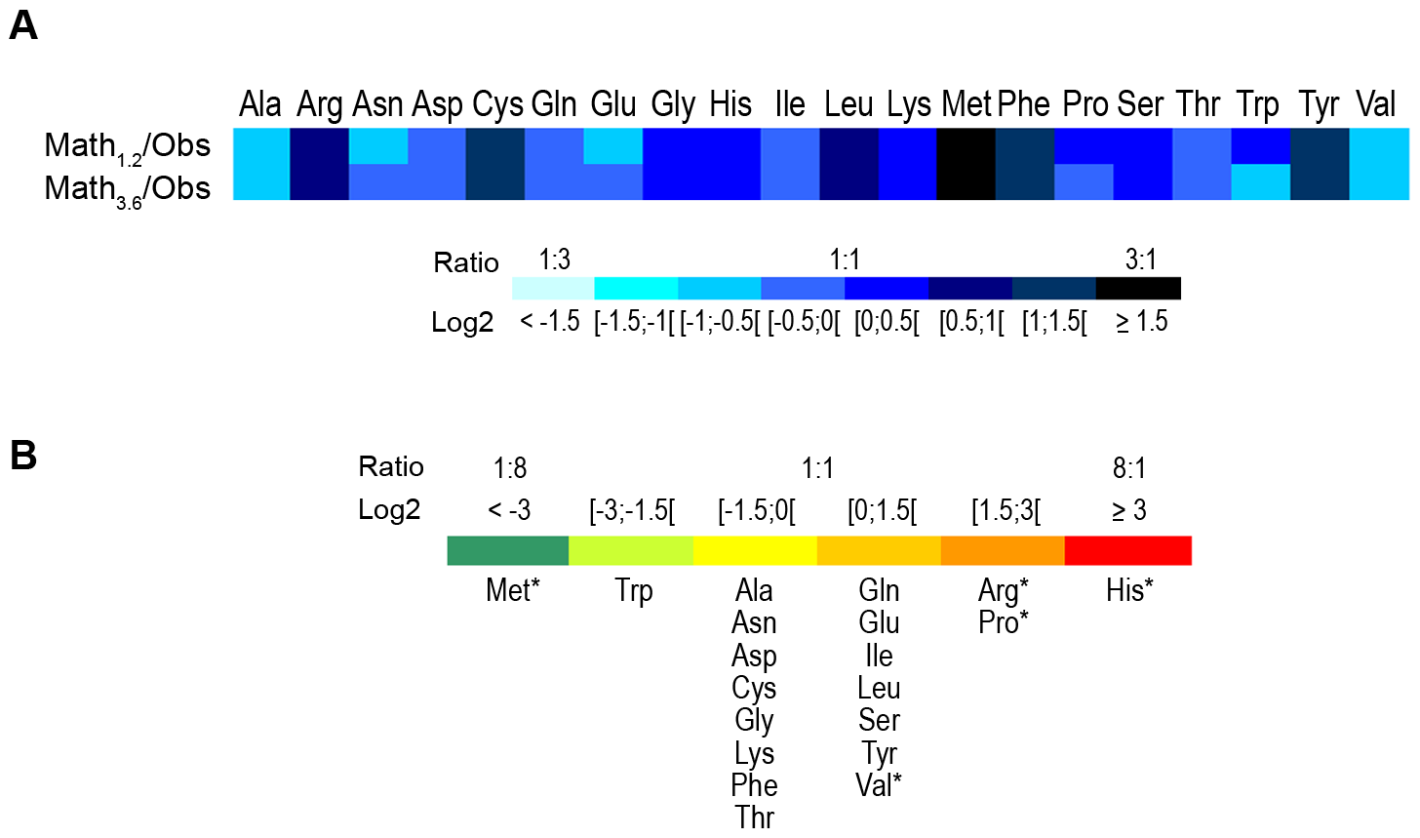
Evolutionary dynamics of amino acid gains and losses. (A) Ratio between the amino acid ‘expected’ frequency (calculated by mathematical modeling using two different overall mean R values, 1.2 (upper row) and 3.6 (lower row) – see methods) and the ‘observed’ frequency for the 251 genetic variants. Normalized ratio intervals (log2) are color-coded. For Trp, which was the unique amino acid that was never created from the total 511 variable sites found in *ompA*, the ‘observed’ frequency was calculated by determining the mid-point of the respective exact 95% confidence interval using the ration between the F and Binomial distribution [57]. (B) Empirical data showing the ratio of created/removed amino acid substitutions that were ‘observed’ among the 251 genetic variants. Normalized ratio intervals (log2) are color-coded, and each amino acid is shown below to the correspondent interval. Statistical significance (*P*≤0.02) was found for the amino acids marked with an asterisk. For panels (A) and (B), logarithmic transformation of the ratios is required for symmetrical distribution of the data around zero.

Considering the above evidenced strong selective pressure acting on this dominant chlamydial antigen, we examined the mutational trend of amino acid gains and losses in MOMP evolution. Therefore, for each prototype strain, a mathematical approach was used to estimate the ‘expected’ frequency of each amino acid considering all possible single nucleotide polymorphisms (SNPs) for each codon position. Depending on genotype, about 3550 different SNPs may randomly occur in *ompA*. Curiously, no significant differences were found for the amino acid frequencies calculated using different overall mean ratios of transitions rate to transversions rate (R). The evolution of the amino acid composition of MOMP was determined by comparing these ‘expected’ frequencies (mathematical approach) with the ones ‘observed’ for all types of genetic variants isolated worldwide (Figure 5A). The amino acids Ala, Val, Thr and Ser were the most accrued in all scenarios, while Trp and Met revealed the lowest frequencies. However, frequency discrepancies were found for Met, Phe Tyr and Cys, suggesting that evolution of amino acid composition in MOMP may be ruled by intrinsic trends that emerge from specific mutational and selective pressures. For example, despite Met is one of the less frequent amino acids, it is being acquired up to 4 times less than the mathematically expected. Also, the observed frequencies for Phe, Tyr and Cys are about 2 times less than the expected. Interestingly, these amino acids were found to belong to the top five ‘gainers’ (His, Arg, Pro, Tyr and Val) or ‘losers’ (Met, Trp, Phe, Cys and Asp) among the worldwide genetic variants, as they presented the highest or the lowest ratios of create/remove substitutions and thus are being evolutionary accrued or lost in MOMP, respectively (Figure 5B). For instance, for each His that is lost, nine are being acquired (*P* = 0.007), while for each Met accrued, 10 are being removed (*P* = 0.01). In contrast to what is traditionally assumed, this nonrandom mutational dynamics suggests that the amino acid composition of MOMP is far from a total equilibrium. Although the impact of these mutational trends in MOMP evolution is not known, the gain or loss of certain amino acids were already shown to influence the structure of several bacterial porins [12]. In agreement with this, we found that about two thirds of the 83 substitutions involving the negatively charged residues Asp and Glu occurred in MOMP external loops, where they are known to be involved in the binding to the lipopolysaccharide [31]. Bioinformatically, these substitutions did not reveal significant differences in protein chemical properties, suggesting that only minor porin structural alterations are expected on behalf of adaptive environmental changes. Also interesting was the preservation of the seven Cys residues, which are 100% conserved among all *C. trachomatis* genotypes. More, the apparent fixation of the CDI Val-to-Cys change in all B variant specimens (isolated from 12 distinct geographic regions) indicates that this Cys residue, which is conserved among the remaining genotypes, is important at this specific protein position. Considering their ability to form intra- and intermolecular disulfide bonds, the conservation of a higher number of Cys residues in MOMP reinforces their role in maintaining the membrane structural integrity of a pathogen that lacks the typical peptidoglycan layer found in other gram-negative bacteria [32].

## Discussion

The detailed understanding of the evolutionary pathway of pathogen's antigens is of the utmost importance, especially when no efficacious preventive strategies (such as a vaccine) are available yet. In this worldwide survey, we intended to provide a more complete picture of the mutational trends of the *C. trachomatis* leading multi-subunit vaccine candidate.

Contrarily to other chlamydial loci, where polymorphism reflects genotype's adaptation to different biological niches and ecological success [21], no compelling association was established at molecular, phylogenetic or immunological level for MOMP. Our data revealed that amino acid composition of MOMP is far from a detailed equilibrium as certain residues seem to be consistently accrued (His, Arg, Pro, Tyr and Val) or lost (Met, Trp, Phe, Cys and Asp) in protein evolution (Figure 5B). This MOMP substitution pattern diverge from the ongoing changes of amino acid frequencies of the remaining *C. trachomatis* chromosome, where Cys, Phe, Arg, Met and His as well as Lys, Leu, Glu Pro and Tyr appear as the five strong gainers and losers, respectively [33]. More, the frequency fluctuations relative to mathematical modelling (Figure 5A) corroborate the singular nature of this key antigen, ruled out by specific mutational and selective pressures. They likely drive protein evolution towards better antigenic fitness, as amino acid changes were almost threefold more frequent in B-cell antigenic regions (with 25% likely at fixation worldwide) than in the remaining protein (*P*<10^−5^), similar to that previously found in Portuguese isolates [23]. Although the *in vivo* implications of these alterations in MOMP are still unknown, some of them were previously shown to prevent recognition and neutralization by host antibodies *in vitro* [7], [8], [27]. Moreover, this immunodominant protein may be strategically used as reservoir for antigenic variability in a chlamydial decoy-like host immune evasion, as MOMP was found to block the binding of more broadly protective species-common pan-neutralizing antibodies, which have been suggested as potential alternatives for a chlamydial vaccine [17].

It has been argued that both the immunogenicity and adhesion role of MOMP may also depend on the protein native conformation (nMOMP) (still unknown) [34]. Consonant with this, we have found a nonrandom mutational dynamics involving amino acids (ex. Asp, Glu and Cys) that are thought to be critical for the nMOMP structure [12], [35], which may consequently affect the conformation and immunoaccessibility of antigenic regions. Recently, by using a nonhuman primate trachoma model, Kari *et al.* [16] found that immunization with the trimeric nMOMP yielded striking different neutralizing antibody titers against two genotype A strains that differ solely by four amino acids in MOMP. Although none of these changes fall within known B-cell epitopes, it suggests that any apparently minor alteration may influence the nMOMP structure [16]. Accordingly, we speculate that the 18 nonsilent alterations occurring outside known VD antigenic regions that seem to be at fixation worldwide (Figure 2), may have important conformational immunogenic implications in nMOMP, with likely more impact in exposed B-cell epitopes than in the transmembrane linear T-cell epitopes.

It has been shown that humoral immune responses facilitate antibody-dependent cellular cytotoxicity and, most importantly, boost the induction of an optimal memory Th1 response through rapid uptake, processing and presentation of antigens [36]. Thus, it is expected that evolutionary fixation of mutations in MOMP B-cell antigenic regions may have pathological consequences at the concertedly humoral and cellular immune responses, which may contribute to chlamydial recurrence or persistence, as previously demonstrated for several virus [37]. In MOMP, CTL and Th epitope clusters of diverse HLA specificities are localized in nonpositively selected CDs (Figure 4), which could apparently constitute promising components for a successful multi-subunit vaccine, enabling widespread protective immunization for a genetically diverse human population [6]. An interesting candidate is the MOMP CDIV species-specific cluster of five CTL and six Th epitopes binding three HLA class I and four HLA class II allotypes [28], [29], in which variability is thought to disrupt vital protein functions and greatly reduce pathogen fitness [6]. This is supported by our data as none of the nonsynonymous mutations occurred in these cluster epitopes seems to become fixed (as shown by the low dN/dS value for this region – see Figure 4A). However, this lack of antigenic variation on these MOMP T-cell clusters might indicate a pathogen's evolutionary mechanism that may lead to immune subversion, analogous to the one found for *Salmonella enterica* serovar Typhimurium [38] and *Mycobacterium tuberculosis* [39], which show a similar intracellular life-style. It is believed that both recognition and consequent cellular immune response to these epitopes might actually provide a net benefit to the pathogen, while may be detrimental to the host [39], as they may maximize the likelihood of pathogen's transmission or persistence. Reinforcing this, it has been shown that MOMP alone is unable to elicit a Th1 response (predominantly by IFN-γ secretion) sufficiently strong to resolve chlamydial infection and confer protective immunity [4]. Thus, it could be speculated that this pathogen may have developed ways of avoiding IFN-γ mediated effectors, clearly compromising the Th1-way of reaching effective protection, which seems mandatory for a vaccine against *Chlamydia*.

Intriguingly, a high level of conservation of MOMP seems to be associated with a higher ecological success, as we have previously suggested [23]. Indeed, the two most prevalent *C. trachomatis* genotypes, E and F, showed an *ompA* mutation rate 22.3-fold lower than that of the other genotypes (*P*<10^−20^). As the existence of distinct antigenic profiles among genital genotypes was previously demonstrated [29], [30], [40], we speculate that the about 12% MOMP amino acid differences among genotypes [21] may confer E and F antigenic profiles better fitted to deal with the host immune system, which would be less prone to change in the light of the Darwinian evolutionary theory. Considering the uniqueness of the chromosomal genetic make-up and evolutionary course of these two genotypes [21], we speculate that E/F specific virulence factors may also contribute for their ecological success, which does not seem to be correlated with the intracellular multiplication rate [41].

In *C. trachomatis*, no weight has usually been given to silent mutations that take place in *ompA*, although they were found to constitute 49.3% and 18.5% of all mutations observed in CDs and VDs, respectively, and some of them seem to be fixed worldwide within some genotypes (Figure 2). However, it has been demonstrated that the generation of synonymous codons may influence the mRNA structure [42], the translation efficiency [43], and the protein folding and tertiary structure [44]. For example, it was shown that some highly expressed genes of *E. coli* [45], *S. cerevisiae* [46] and *B. subtilis* [47] have a strong preference for codons recognized by the most abundant tRNA species, which promotes translational efficiency. Similarly, we have found that 75% of all silent mutations that are likely at evolutionary fixation resulted in synonymous codons with high frequency usage in *C. trachomatis* [25]. This “travelling without moving” phenomenon [48] permit the immediate MOMP adaptive landscape to differ among strains without changing its sequence, function or fitness, allowing the protein to modify its evolutionary pathway according to environmental and host immune pressures.

Another interesting but cautious consideration about beneficial or deleterious mutations in *ompA* relates to the “hitchhiking effect”, where an evolutionarily neutral or even deleterious mutation may spread through the population due to its proximity to a beneficial mutation (being dragged in the same recombinant fragment) [49]. This effect may be expected to occur in *ompA* due to its well-described recombinant character [50], [51], and it may explain the high frequency of some apparent neutral mutations, such as the seven silent mutations in close proximity to three potential advantageous nonsynonymous mutations in CDI of numerous B variant specimens from five to 11 geographic regions (Figure 2). One of these favourable mutations was found to provide all B variant specimens with a Cys residue, which is conserved among the remaining *C. trachomatis* genotypes and thus may play an important role in maintaining the membrane structural integrity.

Although the extant variant specimens reported in this worldwide review may have suffered the effects of the immune system of hundreds of different hosts and may represent several thousands of bacterial generations, still, they constitute a flash in the ∼100 million-year chlamydial evolution [52]. Even with this extremely short time-scale (about six decades), our results clearly evidence the existence of intrinsic mutational trends for MOMP, a crucial antigen, adhesin, and porin. We observed the existence of distinct evolutionary scenarios in MOMP B- and T-cell epitopes that may benefit to the pathogen. Despite the hypothetical accumulation of some mutations in prototype strains (that were here used as “baselines” for the clinical variants) due to *in vitro* passage (which was never demonstrated for *Chlamydia*), there is no immune pressure associated with the cell-lines used for their passage. Thus, even if some mutations occur in laboratory prototype strains they are driven by genetic drift, and it is not credible that they could mask the statistically supported B-cell targeted fixation of mutations observed worldwide for clinical strains. This hitherto unrevealed extensive variation in B-cell antigenic regions suggests a still undisclosed complex scenario of dynamic antigenic profiles for this unavoidable pathogen's antigen, likely representing new neutralization escape mutants that continue to co-evolve with the human host. Together with the apparent conserved T-cell scenario, they likely constitute an obstacle for the development of an efficacious MOMP-based vaccine, whose success will require the presence of other protective antigens and/or the identification of novel broadly cross-reacting conformational-dependent MOMP neutralizing antibodies, whose specificity and protective function are not affected by this mutational scenario.

## Materials and Methods

### Study population

An exhaustive and complete literature search was performed to look for all sequence-based MOMP published surveys containing both variant and nonvariant *ompA* sequences compared to the respective prototype strains. In general, the study population from the 56 surveys performed to date, encompassed patients attending to general practice, family planning, obstetrics, gynaecology and STD clinics, as well as individuals from specific and restrict communities (such as homosexual networks or resource-poor villages where LGV and trachoma are relevant, respectively). To avoid bias potentially arising from the duplication of specimens, the following precautions were taken: i) specimens collected from sexual partners during the same year were excluded; and ii) for longitudinal studies where more than one sample can be collected from the same patient, only one stage of the study was considered as it would be impossible to distinguish if samples of further stages were from re-infection or a merely persistence episode. To our knowledge, none of the strains was propagated in cell culture after a plaque assay (or limiting dilution techniques) in order to obtain clones. Accordingly, one cannot tell whether the determined *ompA* genotype refers to a single clone or to the predominant clone (if a mixed infection occurred).

For genotype comparisons, the traditional prototype strains representing all *C. trachomatis* serovars were used as baseline. Considering that for some prototype strains inconsistencies were observed among *ompA* sequences that were obtained in the 80s and the beginning of 2000 (when automated sequencing by capillary electrophoresis started), only the later were selected because of their likely higher reliability. However, the possibility of these inconsistencies could also be due to different clones of the same prototype strain cannot be discarded. Therefore, the prototype strains used in this study are described in Table 1. Although the use of Ba/Apache2 as reference strain is consensual, it was considered as a B-variant in the present study due to its closer proximity to the prototype B/TW5 than some well-known B strains as well as to its similar *ompA* mutational pattern with several previously classified B-variant strains, which makes any B/Ba-serovar distinction, in our opinion, inaccurate. Moreover, some specimens reported in the literature as D-, I-, and J-variants were found to be identical to prototype strains Da, Ia and Ja, and thus were not considered as variants in the present study.

### Genomic and phylogenetic analyses

Genomic and phylogenetic analyses were performed as previously described [23]. Briefly, for each *ompA* genotype, the MEGA 4.0.2 software (http://www.megasoftware.net) was used to produce alignments, to create matrices of pairwise comparisons, to estimate the number of variable sites, to compute overall mean genetic distances within and between serogroups, and to generate phylogenies at the nucleotide level. For the later, both the Neighbor-Joining method (with Kimura 2-Parameter and Tamura-Nei models) and the Maximum Parsimony method (using the max-min branch-and-bound algorithm) were used as previously described [21]. As more than two-thirds of the mutations are nonsynonymous, these analyses were repeated at the protein level to check the existence of ambiguities. In addition, the mean genetic diversity within each *ompA* genotype-population (π) was estimated using the Maximum Composite Likelihood method. Considering that many genes display a nonrandom usage of synonymous codons for specific amino acids, the SWAAP 1.0.3 software (http://www.bacteriamuseum.org/SWAAP/SwaapPage.htm) was used to compute the codon frequency and the relative synonymous codon usage [46] for all genotypes. Because of the different lengths of the sequences, the pairwise-deletion option was chosen to remove all sites containing missing data or alignment gaps, only when the need arose and not prior to the analyses. Standard error estimates were obtained by a bootstrap procedure (1,000 replicates).

Since recombination is a mechanism of genetic variability, the SimPlot/BootScan software (http://sray.med.som.jhmi.edu/SCRoftware/) was used to evaluate the existence of any recombination event in *ompA* of each specimen, as previously described [53].

### Analysis of molecular evolution

For each *ompA* genotype, the Nei-Gojobori method [54] of MEGA was used to calculate the overall mean of dS and dN, as previously described [55]. The p-distance model was used to normalize the computed differences against the number of potential synonymous and nonsynonymous sites. dS and dN were also evaluated over a sliding window (window size = 15; step size = 3) using the Nei-Gojobori method of the SWAAP. In order to evaluate the statistical significance of the operating selective pressure on MOMP specific domains, a codon-based Z-test of selection was performed to calculate the probability (95% confidence interval) for rejecting the null hypothesis of strict-neutrality (dN = dS) in favor of the positive (dN>dS) or purifying selection (dN<dS). An exhaustive evaluation of the exact codons under positive selection, using a more conservative and powerful bioinformatic approach (maximum likelihood analyses), would required the knowledge of all nonvariant specimens circulating worldwide, which is unfeasible as studies involving solely nonvariant specimens were not included in the present survey.

Since estimation of the ratio of transitions rate (Ts) to transversions rate (Tv) provides insight into the process of molecular evolution of a locus, the Tamura 3-Parameter method [56] of MEGA was used to compute the overall mean of Ts/Tv ratio (R) for all examined *ompA* sequences, while SWAAP was used to evaluate R over a sliding window (window size = 60; step size = 1), in order to identify gene regions in which functional constraints are tolerated.

### Analysis of protein features

To shed some light on the putative impact of each mutation on MOMP, a comparative analysis of the protein sequences of all variant and prototype strains was performed using the Protean program of LaserGene (DNASTAR). Basically, the following protein features were evaluated: charge density, secondary structure, hydropathy, antigenicity, amphiphilicity, surface probability and flexibility. We used a sliding window of a specified range of amino acids with the default parameters of each method, as previously described [23].

### Mathematical modelling

To examine the mutational trend of amino acid composition in MOMP evolution, we used the *ompA* sequences of prototype strains as baseline. We developed a mathematical approach to estimate the ‘expected’ frequency of each amino acid considering all possible SNPs for each position of the ∼397 *ompA* triplets. Depending on genotype, the novel triplets (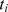) were determined according to the distribution:
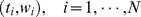
where 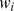 represents the relative weight of each type of SNP. This correction was performed because transitions typically occur in nature much more often than transversions. For comparative purposes, two different overall mean R values were used: one relative to *ompA* (R = 1.2) and the other relative to ∼50 loci of the *C. trachomatis* chromosome (R = 3.6) [21]. Thus, for each possible transition, it was assigned a relative weight of 1.2 or 3.6, while a weight of 1 was assigned for all possible transversions. All amino acids (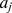) resulting from the novel triplets generated, were determined according to the following distribution:
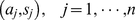
where each 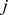 represents the set of indices 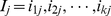 of all triplets 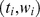 that code a certain amino acid, and
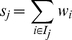
represents the relative weight of each novel amino acid. Considering that one amino acid may be encoded by several synonymous triplets, the relative weight of each amino acid was calculated by summing the weights of all same-amino acid triplets. The final ‘expected’ frequency for each amino acid was calculated according to the formula:
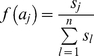
All calculations were performed using the free software environment for statistical computing and graphics R (http://www.r-project.org/).

### Statistic analyses

All statistics was performed using the SPSS Base version 15.0 (SPSS Inc. Chicago) in order to estimate the *P* values by Fisher's exact test as well as the odds ratios with a 95% confidence interval.

## Supporting Information

Figure S1 MOMP antigenic regions. Outline of all B- and T-cell core epitopes mapped for the 17 baseline prototype strains representing the C. trachomatis genotypes.
(0.43 MB PDF)
