# Supplementary material for: Adaptive Evolution of the Chlamydia trachomatis Dominant Antigen Reveals Distinct Evolutionary Scenarios for B- and T-cell Epitopes: Worldwide Survey
Source: PLoS One. 2010 Oct 5;5(10):e13171. doi: 10.1371/journal.pone.0013171 (PMC2950151; doi:10.1371/journal.pone.0013171)
Supplement: Figure S1 — MOMP antigenic regions. Outline of all B- and T-cell core epitopes mapped for the 17 baseline prototype strains representing the C. trachomatis genotypes. (0.43 MB PDF) [file pone.0013171.s001.pdf]

Adaptive evolution of the *C. trachomatis* dominant antigen reveals distinct evolutionary scenarios for B- and T-cell epitopes: worldwide survey

Alexandra Nunes, Paulo J Nogueira, Maria J Borrego, and João P Gomes

Figure S1

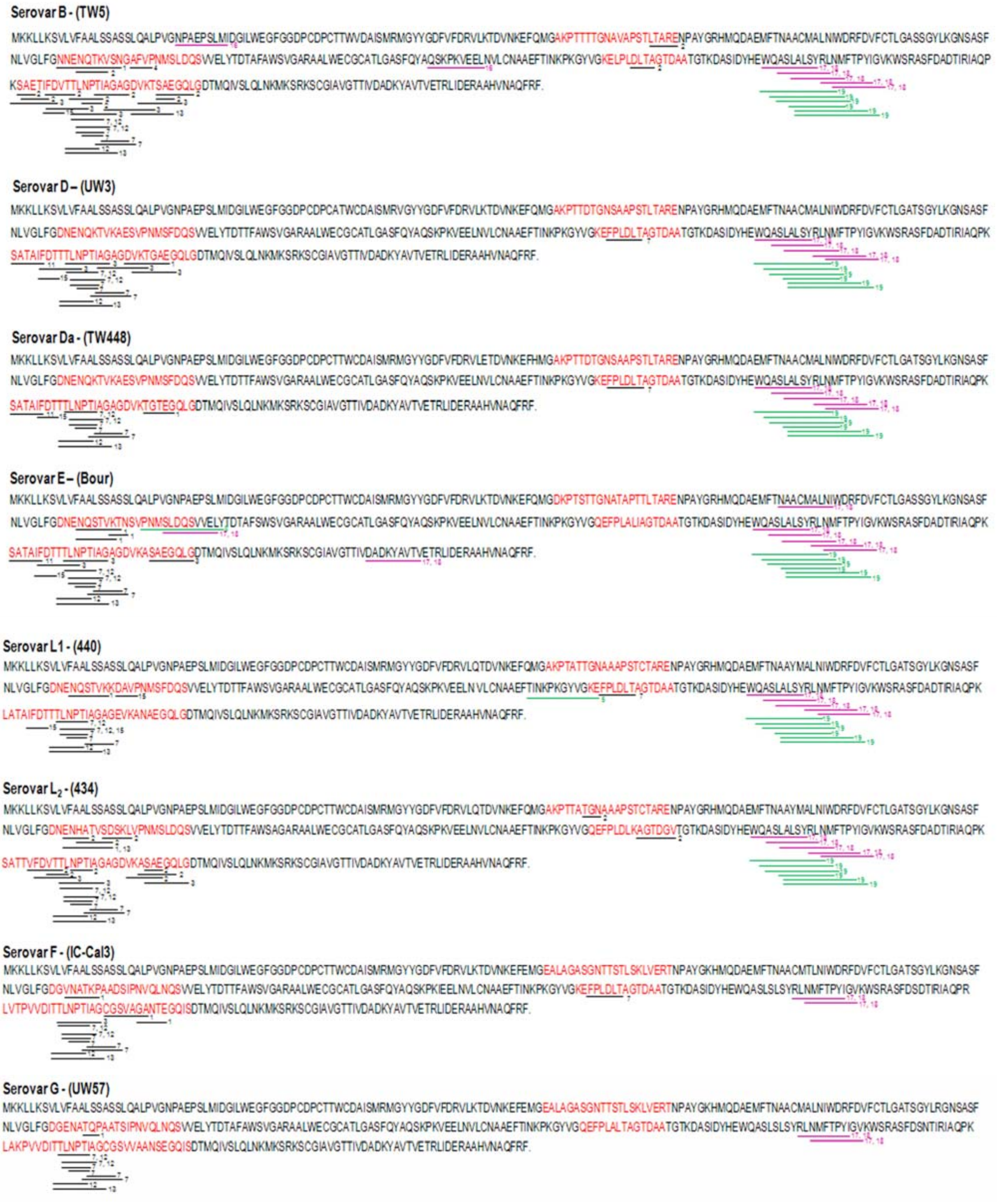

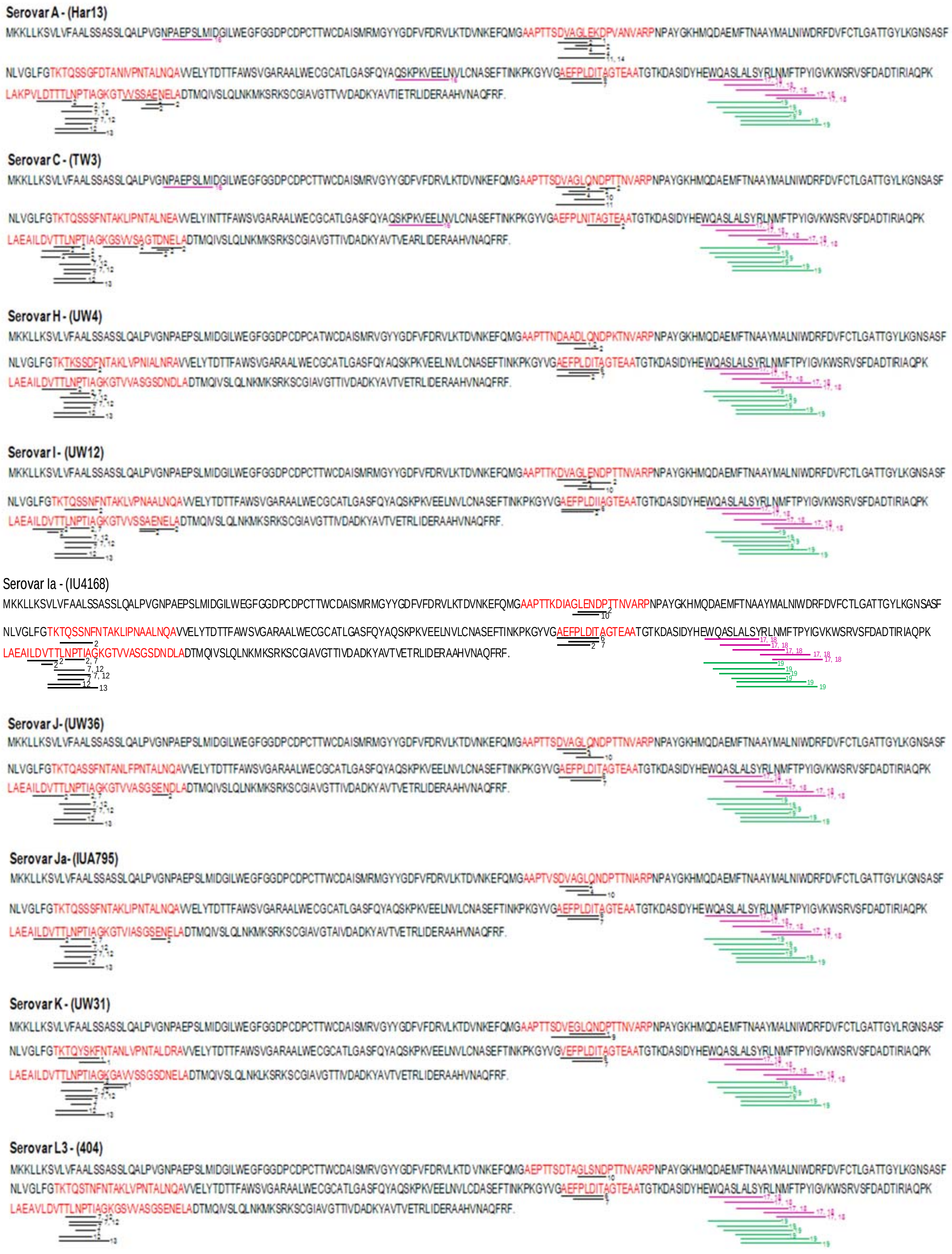

Figure S1. MOMP antigenic regions. Outline of all B- and T-cell core epitopes mapped for the 17 baseline prototype strains representing the *C. trachomatis* genotypes. Amino acid residues belonging to constant domains of the protein are in black, while residues encompassing the variable surface-exposed domains are in red. The B-, CTL-, and Th-cell epitopes are underlined in black, purpure, and

green, respectively. 1- Batteiger BE (1996), Infect Immun 64:542-547; 2- Zhong GM, Brunham RC (1991), Infect Immun 59:1141-1147; 3- Zhong , et al. (1990) Infect Immun 58:1450-1455; 4- Hayes, et al. (1990) J Gen Microbiol 136:1559-1566; 5- Stagg, et al. (1993) Immunol 79:1-9; Pal (1993) J Gen Microbiol 139:1565-1 570; Batteiger, et al. (1996) Infect Immun 64:2839-2841; 8- Arno, et al. (1998) J Infect Dis 178: 1713-1718; 9- Villeneuve, et al. (1994) Microbiol 140:2481-2487; 10- Qu, et al. (1993) Infect Immun 61:1365-1370; 11- Morrison, et al. (1992) In: Sexually transmitted diseases. NY: Raven Press. pp. 57–84; 12- Peterson, et al. (1991) Infect Immun 59:4147-4153; 13- Su, et al. (1990) Infect Immun 58:1017-1025; 14- Su H, Caldwell HD (1992) J Exp Med 175:227-235; 15- Colan, et al. (1988) Mol Microbiol 2:673-679; 16- Holland, et al. (1997) Clin Exp Immunol 107:44-49; 17- Kim, et al. (1999) J Immunol 162:6855-6866; 18- Kim SK, DeMars R (2001) Curr Opin Immunol 13:429-436; 19- Ortiz, et al. (1996) J Immunol 157:4554-4567.
